# Advantages of total proctocolectomy with straight ileoanal anastomosis plus pedicled omental transposition for familial adenomatous polyposis: a preliminary study

**DOI:** 10.1186/s12957-022-02488-3

**Published:** 2022-01-22

**Authors:** Tianci Qin, Jiankun Liao, Haiquan Qin, Linghou Meng, Wentao Wang, Zigao Huang, Jungang Liu, Xianwei Mo

**Affiliations:** 1 Department of General Surgery, Guiping People’s Hospital, No.7, People’s West Road, Guiping, Guigang, 537200 Guangxi Autonomous Region China; 2 Department of Gastrointestinal Surgery, Division of Colorectal and Anal, Guangxi Medical University Cancer Hospital, No.71, Hedi Road, Qingxiu District, Nanning, 530021 Guangxi Autonomous Region China; 3 Guangxi Clinical Research Center for Colorectal Cancer, Division of Colorectal and Anal, Guangxi Medical University Cancer Hospital, No.71, Hedi Road, Qingxiu District, Nanning, 530021 Guangxi Autonomous Region The People’s Republic of China

**Keywords:** Familial adenomatous polyposis, Straight ileoanal anastomosis, Omental transposition, Laparoscopic surgery, Digestive tract reconstruction

## Abstract

**Purpose:**

To achieve excellent postoperative bowel function in familial adenomatous polyposis (FAP) patients, it is important to reconstruct the digestive tract. The aim of this study is to preliminarily discuss the advantages of total proctocolectomy with straight ileoanal anastomosis (TPC-SIAA) plus pedicled omental transposition for FAP.

**Methods:**

A retrospective study was carried out in two hospitals analysing data for FAP patients who underwent surgical treatments between 2015 and 2021. Perioperative outcomes and early and mid-term anal functions were analysed.

**Results:**

After excluding 4 patients who underwent total proctocolectomy with permanent ileostomy, 10 patients were enrolled in the study. Among the 10 patients, 3 received TPC-SIAA plus pedicled omental transposition, 3 received total proctocolectomy with ileal pouch-anal anastomosis (TPC-IPAA), and 4 received total colectomy with ileal pouch-rectal anastomosis (TC-IPRA). Except for one case conversion to laparotomy, laparoscopic surgery was performed for the other cases. The incidence of early postoperative complications was apparently higher with pouch anastomosis (57.1%) than straight anastomosis (0%). Frequencies of bowel movement and low anterior resection syndrome (LARS) score were higher for TPC-SIAA than the other two surgical procedures in the early term; over time, however, the frequencies of bowel movement and LARS score both showed a decreasing trend. In addition, combined with anorectal pressure detection and magnetic resonance imaging defecography at the 3rd month after TPC-SIAA plus pedicled omental transposition, defecation coordination was good. The dynamics and receptivity of the new rectum tended to be as expected.

**Conclusion:**

Although the three surgical procedures are safe and feasible surgical options for FAP, TPC-SIAA plus pedicled omental transposition is more consistent with intestinal physiology, with good intestinal compliance, and anal function tended to be as expected over time. Nevertheless, more extensive studies are needed to confirm these benefits.

## Introduction

Familial adenomatous polyposis (FAP) is an autosomal dominant hereditary disease of the rectum and colon involving hundreds to thousands of adenomatous polyps and extracolonic manifestations characteristics [1, 2]. The incidence of FAP is approximately 1/8300, accounting for approximately 1% of all colorectal cancers. Without early detection and treatment, all or almost all patients will develop colorectal cancer by the age of 40~50 years [3, 4]. Therefore, monitoring and treatment of FAP have become a major focus and difficulty in gastrointestinal disease diagnosis and treatment worldwide [5].

Popular surgical procedures for FAP include total proctocolectomy with permanent ileostomy (TPC-PI) [6], total colectomy with ileorectal anastomosis (TC-IRA) [7], total proctocolectomy with ileal pouch-anal anastomosis (TPC-IPAA) [8], and total proctocolectomy with straight ileoanal anastomosis (TPC-SIAA) [9, 10]. There are pros and cons with each procedure. The decision and timing of FAP surgery have not been standardized, and the choice of surgical method remains a balance between postoperative anal function and radical cure. Therefore, optimizing surgical procedures to improve the long-term bowel function postoperatively of FAP patients is a major goal [11].

In the study of anterior resection syndrome conducted by Dr. Qin [12], the pedicled greater omentum was transposed to the anterior sacral area and filled behind the newly constructed rectum, significantly improving postoperative anterior resection syndrome in patients with low rectal cancer. Indeed, the patients had good postoperative anal defecation and defecation control functions which brought new revelation to us.

In our study, pedicled omental transposition was performed in TPC-SIAA, and we retrospectively analysed data for FAP patients who received previous surgical treatments in two hospitals. We preliminarily discuss the advantages and disadvantages of TPC-SIAA plus pedicled omental transposition for FAP.

## Patients and methods

We retrospectively analysed data for FAP patients who underwent surgical treatments in Guiping People's Hospital and Guangxi Medical University Cancer Hospital between 2015 and 2021. The operations were performed by experienced surgeons. The choice of surgical procedure depended on the prior experience and preference of the surgeon. Patients who received a total proctocolectomy with permanent ileostomy were excluded. The clinical diagnostic criteria of familial adenomatous polyposis are based on colonoscopy revealing > 100 colorectal polyps, mainly adenomatous polyps on pathological examination, with or without family history.

Age, sex, body mass index (BMI), malignant lesion site, surgical procedure, operative time, estimated blood loss, early postoperative complication, length of postoperative hospital stay, and other data were collected and analysed. Measures of the monthly frequency of bowel movement and low anterior resection syndrome (LARS) score [13] within 9 months after surgery were prospectively followed up and comprehensively analysed. If a temporary ileostomy is present, follow-up should begin after closing the ileostomy. In addition, anorectal pressure detection and magnetic resonance imaging (MRI) defecography were used to evaluate new rectal pressure and morphological changes in some patients.

### Surgical procedures of TPC-SIAA plus pedicled omental transposition (shown in Fig. 1)

**Fig. 1:**
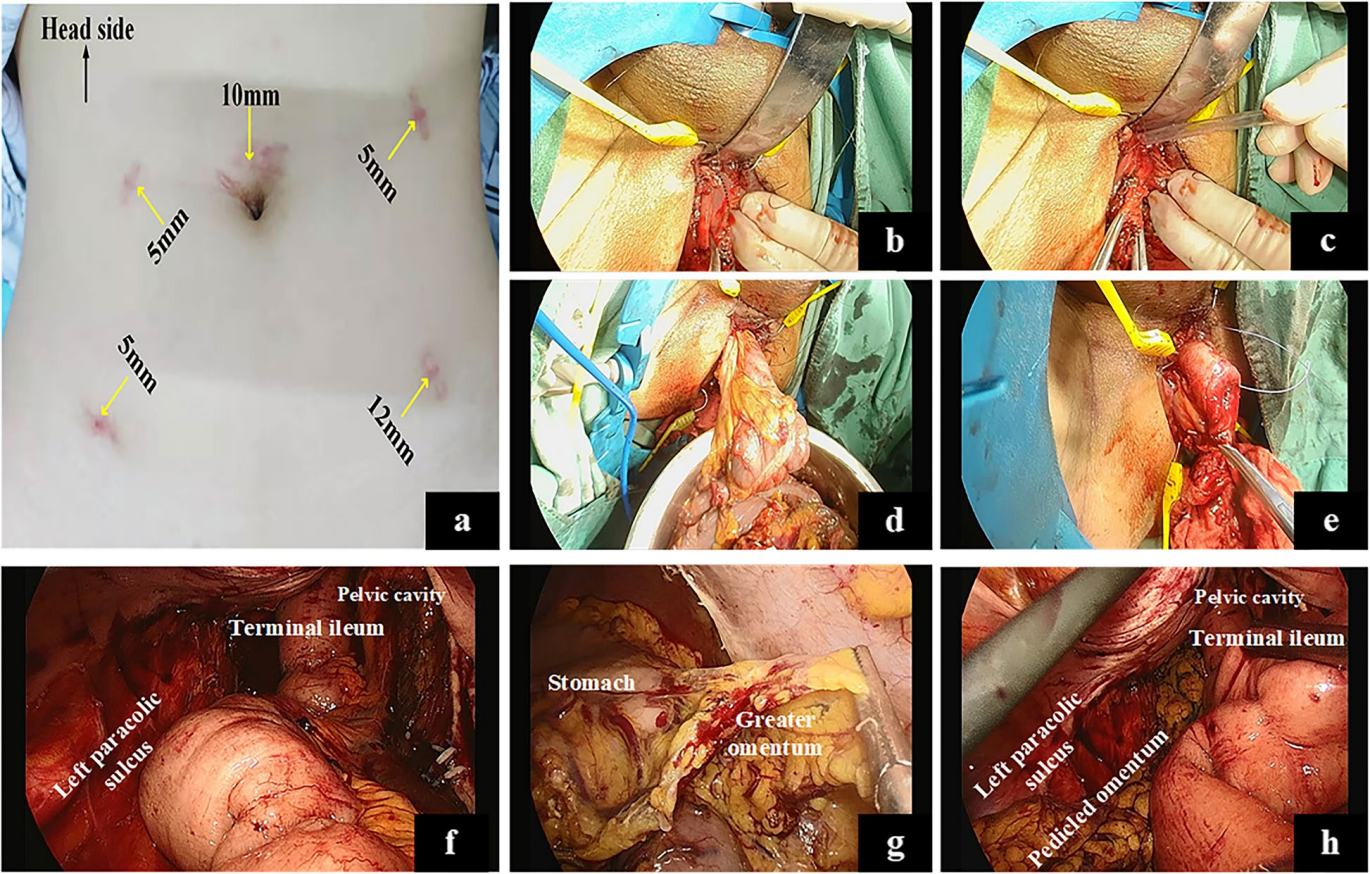
The procedure of TPC-SIAA plus pedicled omental transposition. **a** Trocar placements of total laparoscopic TPC-SIAA plus pedicled omental transposition in a female patient. **b** We transanally stripped the rectal mucosa from the dentate line towards the proximal rectum (black dotted line). **c** We transanally dissociated the rectum to the abdomen-side without damaging the anal sphincter. **d** All rectal and colon specimens were removed through the anus. **e** Straight ileoanal anastomosis was performed through the anus; **f** Laparoscopic pelvic view after straight anastomosis. **g** The pedicled greater omentum was dissociated using a laparoscope. **h** The pedicled greater omentum was filled behind the newly constructed rectum. TPC-SIAA, total proctocolectomy with straight ileoanal anastomosis

#### Abdominal path

The steps are as follows: the right colon, transverse colon, and left colon were dissociated sequentially under laparoscopy. Then the ileum was dissociated to 10-15cm from the ileocecal valve, and its mesentery was cut in order to be removed later. Furthermore, the rectum and its mesentery were dissociated to the anal side as close as possible to the anus, without damaging the anal sphincter.

#### Trans-anal path

At the beginning of the dentate line through the anus, the rectum mucosa was exfoliated cephalad. After exfoliating approximately 2 centimetres upwards, the whole layer of the rectal intestinal wall was cut annularly, and the rectum was dissected. The whole rectum and colon resections were performed by connecting with the transabdominal dissection layer. Then, the free whole rectum, colon, and terminal ileum were pulled out through the anus and removed at the ileum pre-tangent line. The resection of the lesion was completed. Moreover, straight ileo-anal anastomosis was performed manually using intermittent full-thickness suturing.

#### Pedicled omental transposition

The greater omentum was dissociated under laparoscopy, and a pedicled flap of the greater omentum was brought down into the space between the pelvic anterior sacral fascia and the newly constructed rectum. The pedicled omentum and pelvic peritoneum on both sides were fixed with tissue clips to avoid displacing.

### Statistical analysis

IBM SPSS 25.0 (IBM Corp, Armonk, NY, USA) software was used for statistical analysis. Data derived from continuous variables of different groups were compared by the Mann-Whitney *U* test. Categorical data were compared using the chi-squared test and Fisher’s exact test. And the rank-sum test and repeated-measures ANOVA were used for measurement data. A *p* value < 0.05 was defined as statistically significant.

## Results

A total of 14 patients who underwent surgical treatments were included in this study, 10 of whom were enrolled after 4 with TPC-PI were excluded. The median age of the 10 patients was 33 (range 22–68) years, the male to female ratio was 5:5, and the median BMI was 20.83 (14.04–24.84) kg/m2. Among 10 patients, 3 received TPC-SIAA plus pedicled omental transposition, 3 patients received TPC-IPAA, and 4 patients received total colectomy with ileal pouch-rectal anastomosis (TC-IPRA). J-pouch was used in this study. After the specimen was removed through the anus, the terminal ileum of 30 cm was made into J-pouch with two linear cutting closure devices. Except for one case conversion to laparotomy, the other cases were treated with laparoscopic surgery. Table 1 shows a summary of the patients’ characteristics and surgical outcomes.

**Table 1:** Summary of patients’ characteristics and surgical outcomes

Variables	Value(*n* = 10)
Median age [range], (years)	33 [22–68]
Male: female	5:5
Median BMI [range], (kg/m^2^)	20.83 [14.04–24.84]
Type of operation	
TPC-SIAA	3
TPC-IPAA	3
TC-IPRA	4
Median operative time [range], (min)	
TPC-SIAA	385 [260–460]
TPC-IPAA	394 [339–435]
TC-IPRA	412.5 [364–507]
Median EBL [range], (mL)	
TPC-SIAA	100 [100–300]
TPC-IPAA	170 [50–200]
TC-IPRA	175 [50–400]
Median length of postoperative hospital stay [range], (days)	
TPC-SIAA	7 [7–13]
TPC-IPAA	14 [9–38]
TC-IPRA	13 [9–14]
Temporary ileostomy	
Presence	7
Absence	3

*BMI* body mass index, *kg* kilogramme, *m* metre, *TPC-SIAA* total proctocolectomy with straight ileoanal anastomosis, *TPC-IPAA* total proctocolectomy with ileal pouch-anal anastomosis, *TC-IPRA* total colectomy with ileal pouch-rectal anastomosis, *min* minute, *mL* millilitre

### Surgical outcomes

Among all patients, only the 3 who received TPC-SIAA plus pedicled omental transposition did not undergo temporary ileostomy. No early postoperative complication occurred with TPC-SIAA. Regarding TPC-IPAA, 1 patient had bacterial infection and ascites and 1 anastomotic leakage. For TC-IPRA, 1 patient had anastomotic leakage and 1 incomplete ileus. All complications, including anastomotic leakage, were recovered after conservative treatment with intensive anti-infective and protein supplementation, and none of the patients died due to postoperative complications. The individual clinical characteristics and postoperative data of the patients are shown in Table 2.

**Table 2:** Individual clinical characteristics and postoperative data of patients

Case	Age (years)	Gender	Location of cancerization	Type of operation	Temporary ileostomy	Stoma reversal (months)	Early complication	Late complication	Follow-up (months)
1	48	M	Ascending colon, rectum	TPC-SIAA	No	–	No	No	9
2	24	F	No	TPC-SIAA	No	–	No	No	9
3	68	M	Rectum	TPC-SIAA	No	–	No	No	6
4	27	F	No	TPC-IPAA	Yes	11	Bacterial infection, ascites	No	9
5	34	M	Rectum	TPC-IPAA	Yes	3	Anastomotic leak	Constipation	9
6	28	M	No	TPC-IPAA	Yes	5	No	Constipation	9
7	22	F	Descending colon	TC-IPRA	Yes	3	No	No	9
8	54	M	Transverse colon	TC-IPRA	Yes	4	Anastomotic leak	Constipation	9
9	32	F	No	TC-IPRA	Yes	5	No	Constipation	9
10	35	F	Rectum	TC-IPRA	Yes	5	Incomplete ileus	No	9

*M* male, *F* female, *TPC-SIAA* total proctocolectomy with straight ileoanal anastomosis, *TPC-IPAA* total proctocolectomy with ileal pouch-anal anastomosis, *TC-IPRA* total colectomy with ileal pouch-rectal anastomosis

In the group of comparison between pouch and straight anastomosis (shown in Table 3), there were no differences between the two groups in terms of operative time, estimated blood loss, length of postoperative hospital stay, or early postoperative complication (*p*>0.05). However, temporary ileostomy was not performed with straight ileo-anal anastomosis, and no early postoperative complication occurred. Overall, the incidence of early postoperative complication was apparently higher with pouch anastomosis, with anastomotic leakage being the most common early postoperative complication.

**Table 3:** Statistical analysis of different anastomosis types

	Pouch anastomosis (*n* = 7)	Straight anastomosis (*n* = 3)	*p*
Operative time (min)^a^	409.14 ± 56.56	368.33 ± 101.04	0.732
Estimated blood loss (mL)^a^	174.29 ± 118.16	166.67 ± 115.47	0.908
Postoperative hospital day (day)^a^	15.71 ± 10.08	9.00 ± 3.46	0.082
Temporary ileostomy^b^			
Presence	7	0	0.008
Absence	0	3	
sEarly complication^b^			
Presence	4	0	0.200
Absence	3	3	

*min* minute, *mL* millilitre

^a^‾x ± s. Data derived from continuous variables of different groups were compared by the Mann-Whitney *U* test

^b^Categorical data were compared using the chi-squared test, Fisher’s exact test

### Follow-up outcomes

**Fig. 2:**
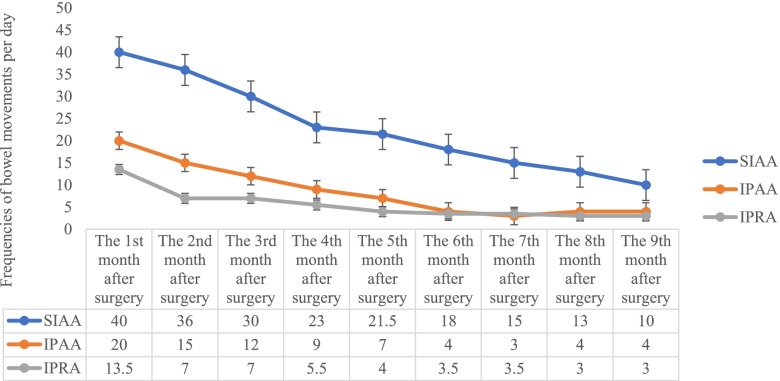
Monthly postoperative median frequencies of bowel movement in the grouping patients (*p* = 0.007 which was using the Bonferroni correction)

**Fig. 3:**
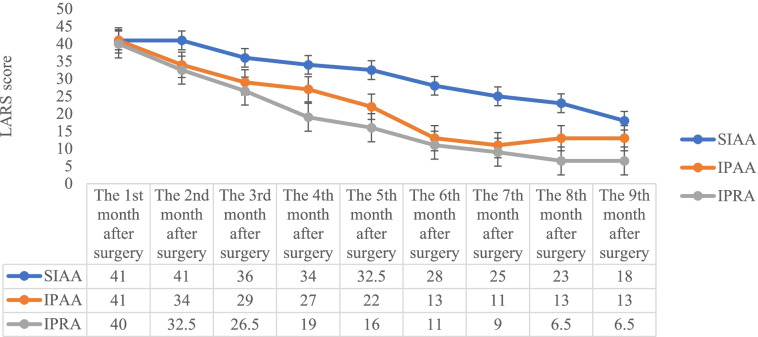
Monthly postoperative median LARS scores in the grouping patients (*p* = 0.055 which was using the Bonferroni correction)

Postoperative follow-up was completed in 9 of 10 patients and at 6 months in one case (shown in Table 2). The monthly postoperative median frequencies of bowel movement are presented in Fig. 2. The frequencies of bowel movement with TPC-SIAA were significantly higher than those with the other two surgical procedures within 9 months postoperatively (*p* = 0.007), but they had a significant downward trend 3 months postoperatively. LARS scores were not significantly different among the three surgical procedures (*p* = 0.055), but LARS scores decreased less with TPC-SIAA than the other approaches (shown in Fig. 3). Although the patients who underwent TPC-SIAA had frequencies of bowel movement early after surgery, there were no cases of anal incontinence. None were readmitted for electrolyte and/or nutritional disorders. The frequencies of postoperative bowel movement and LARS scores gradually decreased over time, self-control ability returned to normal, and the patients were able to return to normal life and work. In long-term follow-up of TPC-IPAA and TC-IPRA, 4/7 patients often had constipation symptoms such as a long toilet time (10–20 min) and a sense of restlessness after defecation.

### Anorectal pressure detection

Anorectal pressure detection was performed in case 1 and case 2 at the 3rd month postoperatively. The data are provided in Table 4.

**Table 4:** Results of anorectal pressure test in case 1 and case 2 at the 3rd month postoperatively

	Case 1	Case 2
Anal canal contraction reflex (KPa)	0	0
Anal canal reflex diastolic pressure (KPa)	1.5	2.2
Anal canal maximum systolic pressure (KPa)	19.2	9.2
The longest contraction time of anal canal (s)	14	23
Anal canal defecation diastolic pressure (KPa)	-2.2	-1.9
Rectal systolic pressure (KPa)	4.6	3.3
Anal canal resting pressure (KPa)	5.8	9.5
Rectal resting pressure (KPa)	0.1	0.2
Functional length of anal canal (mm)	27	27
Rectal sensory capacity threshold (mL)	25	40
Maximum rectal tolerance capacity (mL)	60	100
Action correlation	Negative	Negative

*KPa* kilopascal, *mm* millimetre, *mL* millilitre, *s* second

### MRI defecography

**Fig. 4:**
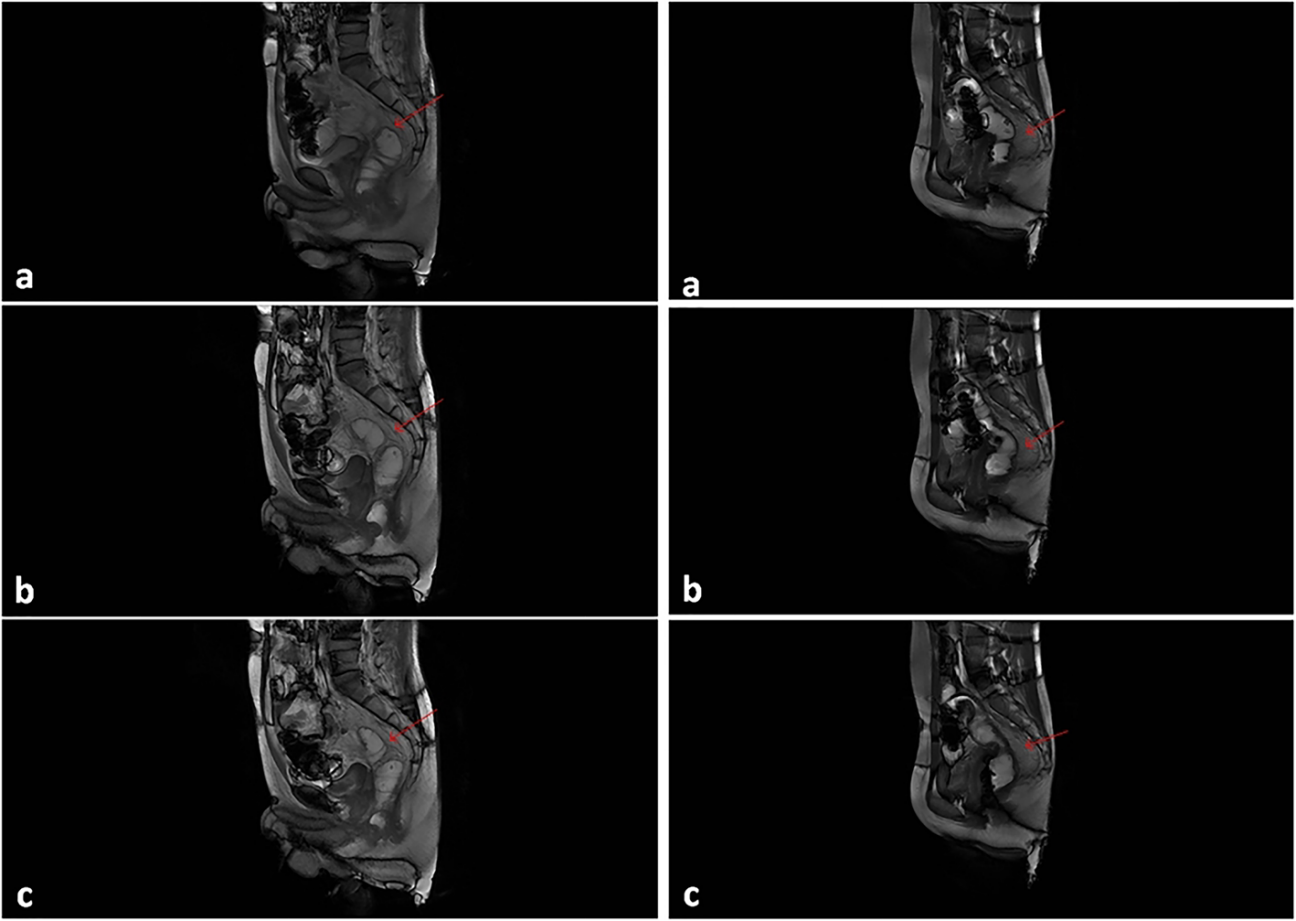
Defecography of two patients at the 3rd month postoperatively (a → b → c shows the continuous movements of defecation). The new rectum showed good dilatation and contraction, and intestinal peristalsis was smooth. Moreover, with peristalsis of the intestine, the reticulum grafted behind the intestine (shown by the red arrow) was well deformed, helping to improve intestinal compliance

The relationship between movement of the transposed omentum and the new rectum was evaluated by MRI defecography in case 1 and case 2 at the 3rd month postoperatively. After the coupling agent was injected via anal intubation, the patient was asked to complete the actions of reposing, elevating the anus, and forcibly defecating; dynamic scanning was completed sequentially during the above steps. The high-resolution T2WI sequence (transverse, coronal, and sagittal view) of rectal MRI showed no definite abnormality in the supra-anal distance; the anorectal angle was enlarged, the puborectalis muscle impression became deeper during forcibly defecating, and no actual signs of mucosal prolapse were observed. The distance between the sacrum and rectum (measured by plane of S4 cone) was close to the preoperative level in the reposing, elevating the anus, and forcibly defecating phases. Moreover, no clear abnormality in sacral coccygeal curvature or pubococcygeal line was observed. In the dynamic images, the presacral transposed omentum oscillated obviously with the peristalsis of the new rectum during the phase of elevating the anus and forcibly defecating. And the overall bowel mobility and intestinal cavity flexibility were good. The coupling agent was discharged smoothly, and the intestinal dynamics were close to those of control individuals during defecation. Continuous MRI defecography images of the two cases are shown in Fig. 4.

## Discussion

Patients diagnosed with FAP are suggested to undergo early radical or prophylactic surgery, with total rectum and colon resection being the most recommended surgical concept today [14]. However, some centres have reported that young patients with less rectal adenomas or a strong desire to have children choose total colon resection and retain part of the rectum [15]. Although studies have shown that postoperative functional recovery is optimal, there is an approximately 10% chance of recurrence and malignant transformation with the residual rectum [16, 17], and the risk of residual cancer increases with age [18]. For recurrent adenomas, the necessity of rectal resection has not been determined. Some studies have confirmed the success rate of endoscopic resection, but the long-term efficacy of endoscopic therapy remains to be proven due to the high risk of recurrence [19, 20]. Proctectomy is radical and advisable for patients without special requirements. Moreover, for high-risk patients with FAP, one-stage total rectal and colon resection is a radical surgical option worthy of priority.

The choice of surgical methods for FAP depends largely on the experience of each clinical centre and the preference of the surgeons. TPC-IPAA has the advantages of both a thorough operation and anal preservation. Nevertheless, the technical requirements of this surgical method are high, as is the risk of postoperative complications, especially pouchitis, anastomotic leakage, and constipation, among others [8, 21], which often affects postoperative quality of life. Although previous studies [22, 23] have confirmed that TPC-SIAA is as safe and feasible as TPC-IPAA, it is seldom selected by surgeons because of the higher frequencies of postoperative bowel movement. However, during TPC-SIAA in our study, the whole rectum and colon were completely dissociated, then directly pulled out through the anus, and removed manually, followed by straight ileoanal anastomosis. This is more consistent with intestinal physiology, without making a pouch or using expensive linear cutter stapler or circular stapler, rendering the procedure easy and less risky, with operative cost savings.

According to the groups of pouch anastomosis and straight anastomosis, straight anastomosis is better than pouch anastomosis in the length of postoperative hospital stay and incidence of early postoperative complication, but there was no significant difference, which may be due to the small sample size. In general, pouch anastomosis appeared to require longer operation times owing to the pouch preparation. Of high importance is the occurrence of early postoperative complication, which influences the length of postoperative hospital stay. Due to the wide range of pouch anastomoses, anastomotic leakage is still the most common early postoperative complication. Compared to straight anastomosis, the technical requirements for pouch anastomosis are higher, but there is a risk of secondary intestinal resection due to pouch failure intraoperatively [24, 25]. Pouchitis is one of the most common postoperative long-term complications, which seriously affects quality of life and may even result in the requirement of a second surgery [26].

Recovery of postoperative bowel function is an important issue after total rectum and colon resection, with reconstruction of the digestive tract being the most important. Straight ileoanal anastomosis and J-pouch ileoanal anastomosis are commonly used for digestive tract reconstruction [14]. The rectal ampulla is removed by surgery, and the patient loses the function of temporary storage of faeces, leading to frequent bowel movements after surgery. Through follow-up, it was found that for straight anastomosis, patients with pouch anastomosis had lower frequencies of bowel movement in the early term, which was due to the storage of faeces by artificial bags. However, it was also found that some patients who underwent pouch anastomosis experienced constipation in the long term, which was considered to be related to the opposite direction of peristalsis of the two intestinal canals used to produce the ileum pouch, violating the principle of pro-peristalsis. In straight ileoanal anastomosis, the frequencies of bowel movement and LARS scores decreased after 3 months postoperatively. This may be related to the terminal ileum, namely, the new upper rectum, expanding compensatively, resembling the “rectal ampulla,” and re-establishing the function of faeces storage. At the same time, the intestinal canal under straight ileoanal anastomosis undergoes cis peristalsis, which is more in line with intestinal physiology.

On the other hand, poor bowel function postoperatively is associated with severe pelvic floor tissue adhesion and poor intestinal compliance after digestive tract reconstruction [27]. With a second operation in the pelvic cavity, a new organizational structure with severe scar adhesion and the mesangial adipose replaced with scar fibrous tissue can be observed in the rectum and pelvic floor, which makes the new rectum stiff. This leads to decreased dilation and poor compliance of the new rectum; even a tiny amount of faeces can cause increased intestinal pressure and frequent occurrence of stools [28]. A previous study found that by using pedicled omental transposition to fill the pelvic floor, reconstruct the presacral structure, increase the intestinal buffer force, and improve compliance of the new rectum, patients have better postoperative stool control function than a control group [12]. In addition, pedicled omental transposition is a simple and easy method with a short operative time. In our study, pedicled omental transposition was found to be safe and feasible for TPC-SIAA. It stimulated reconstruction of the mesenteric fascia, increased the thickness of the presacral space, avoided the intestinal stiffness caused by adhesion between the new rectum and the presacral tissue, cushioned intestinal peristalsis, improved intestinal compliance, and helped to improve postoperative bowel function.

Through postoperative follow-up, it was found that patients receiving TPC-IPAA and TC-IPRA had lower frequencies of bowel movement and faster LARS score recovery in the early term, but that patients receiving TPC-SIAA plus pedicled omental transposition had a higher frequencies of early bowel movement. However, over time, especially after 3 months, defecation improved significantly, like TPC-IPAA. Self-control was also restored without constipation symptoms. We consider that this is related to compensatory dilatation of the new rectum, the paving effect of better omental transposition, and the recovery of defecation control. The LARS score includes intense subjectivity and prominent human interference factors, and the evaluation of postoperative anal function is not comprehensive and objective. For this reason, we combined anorectal pressure measurements in patients who received TPC-SIAA plus pedicled omental transposition at the 3rd month postoperatively. We found that the anal canal resting pressure in 2 patients was far greater than the rectal resting pressure. When there was no stool or a small amount of seat in the new rectum, the patients were able to control defecation without faecal incontinence. At the same time, anal canal contraction can help in control, whereby rectal resting pressure increases only after the stool volume increases and the patient needs to defecate. Moreover, defecation coordination was good, and the rectoanal inhibitory reflex returned to normal. Three months after surgery, MRI defecography also showed that the new upper rectum of the above two patients had good intestinal dilatation, smooth intestinal peristalsis, and overall coordinated defecation. In addition, the transposed omental deformation was good, and intestinal compliance was high. The dynamics and receptivity of the new rectum tended to be as expected.

The necessity of establishing a temporary ileostomy after digestive tract reconstruction is still not clear [29], and it has been reported that it is mostly used to prevent anastomotic leakage after J-pouch ileoanal anastomosis or as a means of rescue after anastomotic leakage [30, 31]. In our study, temporary ileostomy was performed in both TPC-IPAA and TC-IPRA, but not TPC-SIAA plus pedicled omental transposition, and there were no early complications in TPC-SIAA plus pedicled omental transposition. The end of the ileum is straight anastomosed with the anal canal, and the anastomotic site is established at the anal orifice. We believe that there is no need for concern about the risk of anastomotic leakage; thus, prophylactic ileostomy is not needed, which can avoid second-stage closure of ileostomy, as well the inconvenience and inferiority caused by abdominal wall stoma and maximize cosmetic treatment.

Although the anal function of patients who received TPC-SIAA plus pedicled omental transposition gradually recovered at 3 months after surgery, the results of frequent bowel movements within the short term after the process could not be ignored. Disorder in postoperative bowel function causes frequent defecation, causing serious effects on patients. In addition, long-term stimulation of intestinal contents around the anus would cause perianal inflammation, and patient discomfort would be more obvious. The patient’s symptoms were significantly reduced within one month by cleaning the perianal area with warm water after defecation and applying skin ostomy powder, and there were no serious secondary complications.

Due to the extremely low incidence of FAP, there were some certain limitations in this study. The first is related to the small sample size, which leads to bias of statistics and results. Secondly, short-term follow-up results do not fully represent postoperative anal function. Finally, the follow-up contents are general and lack characteristics, and the quality of life is not included in the follow-up index.

In conclusion, TPC-SIAA plus pedicled omental transposition for FAP is safe and feasible. And due to its consistency with intestinal physiology and good intestinal compliance, the recovery of anal function tends to be expected. However, long-term follow-up and prospective studies with a larger sample size are needed to validate the advantages of this approach.

## Data Availability

The data that support the findings of this study are available from the corresponding author upon reasonable request.
